# ﻿*Yamadazymaakebiae* sp. nov. and *Y.hainanensis* sp. nov. (Debaryomycetaceae, Saccharomycetales) from leaf in China

**DOI:** 10.3897/mycokeys.119.156745

**Published:** 2025-07-14

**Authors:** Peng Wang, Chun-Yue Chai, Qiu-Hong Niu, Feng-Li Hui

**Affiliations:** 1 School of Life Science, Nanyang Normal University, Nanyang 473061, China Nanyang Normal University Nanyang China; 2 Research Center of Henan Provincial Agricultural Biomass Resource Engineering and Technology, Nanyang Normal University, Nanyang 473061, China Nanyang Normal University Nanyang China

**Keywords:** Ascomycetous yeast, phylloplane, phylogeny, taxonomy

## Abstract

Members of the genus *Yamadazyma* are widely distributed and frequently found in plant materials. In an effort to investigate the species diversity within this genus, leaf samples collected from Guizhou and Hainan provinces in China were examined. This study led to the discovery of two previously undescribed taxa. Phenotypic examination, combined with phylogenetic analysis based on concatenated ITS and LSU D1/D2 sequences, confirmed their affiliation with *Yamadazyma* and supported their designation as novel species. One of the new species, *Yamadazymaakebiae***sp. nov.**, is phylogenetically closely related to *Y.kanchanaburiensis*, whereas *Y.hainanensis***sp. nov.** represents a distinct lineage, clearly separated from other known *Yamadazyma* species. Detailed descriptions, illustrations, and comparative discussions with their nearest relatives are provided. These findings contribute two additional species to the genus and enhance our understanding of *Yamadazyma* diversity in China.

## ﻿Introduction

The genus *Yamadazyma*, belonging to the ascosporogenous yeasts within the family Debaryomycetaceae, was initially established by Billon-Grand (1989) and later delineated through phylogenetic analyses by Kurtzman and Suzuki (2010). Currently, *Yamadazyma* encompasses 68 recognized species, including 35 anamorphic taxa previously classified within the *Yamadazyma* clade of the genus *Candida* (Khunnamwong et al. 2023; Avesani et al. 2024; Seike et al. 2024). Members of this genus are characterized by several shared phenotypic features, such as the synthesis of coenzyme Q-9 (CoQ-9), the ability to form pseudohyphae, sugar fermentation capacity, and a requirement for external vitamins to support growth (Billon-Grand 1989; Kurtzman 2011). Furthermore, seven teleomorphic species—*Y.akitaensis*, *Y.mexicana*, *Y.nakazawae*, *Y.philogaea*, *Y.riverae*, *Y.scolyti*, and *Y.triangularis*—are known to produce one to four hat-shaped ascospores on sporulation media (Kurtzman 2011; Lopes et al. 2015).

Species of the genus *Yamadazyma* exhibit a cosmopolitan distribution and have been isolated from a wide array of substrates. These yeasts are frequently recovered from plant-associated materials (Lu et al. 2004; Groenewald et al. 2011; Kaewwichian et al. 2013; Lopes et al. 2015; Maksimova et al. 2020; Gao et al. 2021; Khunnamwong et al. 2023), mushrooms (Nakase et al. 2008; Khunnamwong et al. 2023), various food sources (Nisiotou et al. 2010; Avesani et al. 2024), marine environments (Burgaud et al. 2011, 2016), insects (Suh et al. 2005), soil samples (Seike et al. 2024), estuarine water (Am-In et al. 2011), and even the atmosphere (Kurtzman et al. 2001). Nevertheless, a significant proportion of known species are predominantly associated with decaying wood, insects, and insect frass (Groenewald et al. 2011; Kurtzman 2011; Gao et al. 2021). Notably, the genus also includes clinically important species, such as *Y.aaseri*, *Y.conglobata*, *Y.mexicana*, *Y.pseudoaaseri*, and *Y.triangularis* (Kurtzman 2011; Lachance et al. 2011; Al-Sweih et al. 2017; Giachos et al. 2024). In recent years, interest in the ecological roles, biocontrol capacities, and biotechnological applications of *Yamadazyma* has increased. For instance, *Y.cocois*, an endophytic yeast, resides within coconut tissues without causing harm to the host plant (Maksimova et al. 2020). *Y.mexicana* has been shown to suppress phytopathogenic fungi, offering effective control of anthracnose in avocados at both preharvest and postharvest stages (Gonzбlez-Gutiйrrez et al. 2024). A yet undescribed species, referred to as *Yamadazyma* sp. 1, demonstrated the ability to ferment cellobiose, indicating potential for bioethanol production (Lopes et al. 2018). Moreover, *Y.triangularis* exhibits probiotic potential and has been utilized to produce novel antioxidant peptides (Cai et al. 2022). These findings underscore the ecological, agricultural, and biotechnological significance of *Yamadazyma*, highlighting the need for continued and in-depth biodiversity investigations within this genus.

China harbors a high diversity of *Yamadazyma* species, with current records documenting nine taxa reported from various regions across the country (Gao et al. 2021). The first species described from China was *Y.diospyri*, isolated from Kaki fruit by Lu et al. (2004). Subsequently, *Y.paraphyllophila* was identified on phylloplane from Taiwan, China (Kaewwichian et al. 2013). Over the past decade, our research group has conducted extensive taxonomic investigations on *Yamadazyma* in China, particularly focusing on isolates from decaying wood. These efforts led to the identification of seven species, including three previously known taxa—*Y.insectorum*, *Y.akitaensis*, and *Y.olivae*—and four novel species: *Y.dushanensis*, *Y.luoyangensis*, *Y.ovata*, and *Y.paraaseri* (Gao et al. 2021). More recently, *Y.triangularis*, originally described from Japan, was newly recorded in China from dry-cured Xuanwei ham (Cai et al. 2022). Accordingly, China has been identified as a biodiversity hotspot for the discovery of unexpected novel species and newly recorded species of *Yamadazyma*.

Despite recent progress, the species richness of *Yamadazyma* in China remains underexplored. Comprehensive surveys encompassing diverse ecosystems and substrates, particularly in under-sampled regions, are essential to uncover the full extent of the genus’s diversity and to enhance our understanding of its ecological and evolutionary significance. In our most recent investigations conducted over the past two years, several new *Yamadazyma* strains were isolated from plant leaves. Further phenotypic and molecular phylogenetic analysis confirmed that these strains represent two distinct novel species: *Yamadazymaakebiae* sp. nov. and *Y.hainanensis* sp. nov. Their formal descriptions are provided herein, contributing to the expanding inventory of *Yamadazyma* species in China and advancing the taxonomic and ecological framework of the genus.

## ﻿Materials and methods

### ﻿Sample collection and yeast isolation

Living leaf samples were collected from two locations in China: the Guiyang Medicinal Botanical Garden, Guizhou Province (26°34'51"N, 106°42'36"E), and Wuzhi Mountain, Hainan Province (18°17'21"N, 109°40'55"E). Yeast strains representing putative novel species were isolated from the leaf surface using the washing and dilution approach outlined by Jiang et al. (2024). Fresh leaves were cut into small segments using sterile scissors and placed into 10 mL sterile centrifuge tubes containing sterile water supplemented with 0.05% Tween 80. The samples were shaken for approximately 10 minutes, after which the suspension was serially diluted to 10^−2^. A volume of 200 μL from each dilution was spread onto yeast extract-malt extract (YM) agar plates (0.3% yeast extract, 0.3% malt extract, 0.5% peptone, 1% glucose, and 2% agar) supplemented with chloramphenicol (200 μg/mL) to inhibit bacterial growth. Plates were incubated at 25 °C for 5–7 days. Emerging yeast colonies were subcultured on fresh YM agar to obtain pure isolates. Purified strains were suspended in 20% (v/v) glycerol and stored at −80 °C for long-term preservation.

### ﻿Phenotypic characterization

Morphological, physiological, and biochemical characteristics were assessed following standardized protocols outlined by Kurtzman et al. (2011). Colony morphology was evaluated on YM agar after incubation at 25 °C for 7 days. Cellular morphology was observed in YM broth cultures incubated at 25 °C for 3 days using a LEICA DM2500 microscope (LEICA, Wetzlar, Germany) equipped with LAS V4.13 software. To determine the sexual state, single or mixed cultures of each strain were incubated on various sporulation media, including YM agar, 5% malt extract (ME) agar (5% malt extract and 1.5% agar), cornmeal (CM) agar (2% cornmeal and 1.5% agar), Fowell’s acetate agar (0.5% sodium acetate and 2% agar), and Gorodkowa agar (0.1% glucose, 0.5% sodium chloride, 1% peptone and 2% agar). Cultures were maintained at 15 °C and 25 °C for up to six weeks, with observations recorded at two-week intervals (Kurtzman 2011; Gao et al. 2021). Sugar fermentation was examined in liquid medium using Durham fermentation tubes. Carbon and nitrogen assimilation tests were conducted in liquid media, with nitrogen assimilation assessed using starved inocula (Kurtzman et al. 2011). Temperature tolerance was evaluated on YM agar plates at 15, 20, 25, 30, 35, and 37 °C. All newly proposed species names and corresponding descriptions have been deposited in the MycoBank database (https://www.mycobank.org; accessed on 25 February 2025).

### ﻿DNA extraction, PCR amplification, and sequencing

Genomic DNA was extracted from actively growing yeast cells cultivated on YM agar using the Ezup Column Yeast Genomic DNA Purification Kit, following the manufacturer’s instructions (Sangon Biotech Co., Shanghai, China). The ITS region and the D1/D2 domain of the LSU rRNA gene were amplified using primer pairs ITS1/ITS4 (White et al. 1990) and NL1/NL4 (Kurtzman and Robnett 1998), respectively. PCR amplifications were conducted in a 25 μL reaction mixture containing 9.5 μL of nuclease-free water, 12.5 μL of 2× Taq PCR Master Mix with blue dye (Sangon Biotech Co., Shanghai, China), 1 μL of genomic DNA template, and 1 μL of each primer. The thermal cycling protocol consisted of an initial denaturation at 98 °C for 2 minutes, followed by 35 cycles of denaturation at 98 °C for 10 seconds, annealing at 55 °C for 10 seconds, and extension at 72 °C for 15 seconds, with a final extension at 72 °C for 5 minutes. PCR products were examined by electrophoresis on 1% agarose gels. Amplicons displaying clear single bands were purified and sequenced by Sangon Biotech (Shanghai) Co., Ltd. (Shanghai, China). Forward and reverse sequences were edited and assembled into consensus sequences using BioEdit v7.1.3.0 (Hall 1999). Sequence similarity searches were performed using the BLASTN 2.2.19 algorithm (Zhang et al. 2000) against the GenBank database. All newly generated sequences were submitted to GenBank (https://www.ncbi.nlm.nih.gov/genbank/; Table 1).

**Table 1.** Sequences used in molecular phylogenetic analysis. Entries in bold are newly generated in this study.

Species	Strain no.	Locality	GenBank accession no.	
			ITS	LSU D1/D2
*Yamadazymaaaseri*	CBS 1913^T^	Norway	AY821838	U45802
***Yamadazymaakebiae***	**NYNU 22830^T^**	**China**	**OP566868**	**OP566866**
	**NYNU 221015**	**China**	**PV404187**	**PV539463**
*Yamadazymaakitaensis*	CBS 6701^T^	Japan	DQ409164	U45766
*Yamadazymaamphixiae*	CBS 9877^T^	Panama	EU491501	AY520327
*Yamadazymaandamanensis*	CBS 10859^T^	Thailand	AB525239	AB334210
*Yamadazymablattariae*	CBS 9876^T^	Panama	FJ715435	AY640213
*Yamadazymabuinensis*	CBS 6796^T^	Papua New Guinea	HQ283376	U45778
*Yamadazymacerambycidarum*	CBS 9879^T^	Panama	AY964669	AY520299
*Yamadazymaconglobata*	CBS 2018^T^	—	AJ539370	U45789
*Yamadazymadendronema*	CBS 6270^T^	South Africa	HQ283365	U45751
*Yamadazymadiddensiae*	CBS 2214^T^	USA	AY580315	U45750
*Yamadazymadiospyri*	CBS 9769^T^	China	AY450919	AY450918
*Yamadazymadushanensis*	CBS 13914^T^	China	KM272249	KM272248
*Yamadazymaendomychidarum*	CBS 9881^T^	Panama	AY964672	AY520330
*Yamadazymaendophytica*	CBS 14163^T^	Thailand	KT307981	KT307981
*Yamadazymaepiphylla*	CBS 13384^T^	Thailand	LC006082	LC006026
*Yamadazymafriedrichii*	CBS 4114^T^	Germany	HQ283377	U45781
*Yamadazymagermanica*	CBS 4105^T^	Germany	HQ283366	AF245401
*Yamadazymagorgasii*	CBS 9880^T^	Panama	AY964670	AY520300
***Yamadazymahenanensis***	**NYNU 24829^T^**	**China**	**PQ568984**	**PQ568981**
	**NYNU 24905**	**China**	**PV539466**	**PV539465**
	**NYNU 249216**	**China**	**PV539467**	**PV539468**
*Yamadazymainsectorum*	CBS 6213^T^	South Africa	HQ283372	U45791
*Yamadazymajaroonii*	CBS 10790^T^	Thailand	AB360437	DQ404493
*Yamadazymakanchanaburiensis*	CBS 11266^T^	Thailand	NR_137581	KY106534
*Yamadazymakeroseneae*	CECT 13058^T^	UK	FJ235128	FJ357698
*Yamadazymakhao-thaluensis*	CBS 8535^T^	Thailand	HQ283374	HQ283383
*Yamadazymakitorensis*	CBS 14158^T^	Japan	LC060995	LC060995
*Yamadazymakoratensis*	TBRC 14868^T^	Thailand	LC601013	LC601009
*Yamadazymakoratica*	CBS 10789^T^	Thailand	AB360443	AB354232
*Yamadazymalaniorum*	CBS 14780^T^	USA	KY588337	KY588136
*Yamadazymalessepsii*	CBS 9941^T^	Panama	AY964671	AY640214
*Yamadazymaluoyangensis*	NYNU 201023^T^	China	MW365549	MW365545
*Yamadazymamembranifaciens*	CBS 1952^T^	India	AJ606465	U45792
*Yamadazymamexicana*	CBS 7066^T^	Agria cactus	AB054110	U45797
*Yamadazymamolendinolei*	CBS 18660^T^	Italy	PP149061	PP130150
*Yamadazymamichaelii*	CBS 9878^T^	Panama	AY964673	AY520329
*Yamadazymanaeodendra*	CBS 6032^T^	South Africa	AY580316	U45759
*Yamadazymanakazawae*	CBS 6700^T^	Japan	EU343867	U45748
*Yamadazymaoleae*	CBS 18662	Italy	NR_199097	PP375118
*Yamadazymaolivae*	CBS 11171^T^	Greece	FJ715432	FJ715430
*Yamadazymaovata*	NYUN 191125^T^	China	MT990560	MT990559
*Yamadazymaparaaseri*	NYNU 1811114^T^	China	MK682794	MK682805
*Yamadazymaparaphyllophila*	CBS 9928^T^	Taiwan	AY559447	AY562397
*Yamadazymaphilogaea*	CBS 6696^T^	South Africa	AB054107	U45765
*Yamadazymaphyllophila*	CBS 12572^T^	Thailand	AB734050	AB734047
*Yamadazymapseudoaaseri*	CBS 11170^T^	Germany	JN241686	JN241689
*Yamadazymariverae*	CBS 14121^T^	Brazil	KP900044	KP900043
*Yamadazymascolyti*	CBS 4802^T^	USA	EU343807	U45788
*Yamadazymasiamensis*	CBS 12573^T^	Thailand	AB734049	AB734046
*Yamadazymasisaketensis*	TBRC 17139^T^	Thailand	OP811260	OP811260
*Yamadazymasongkhlaensis*	CBS 10791^T^	Thailand	AB360438	DQ404499
*Yamadazymatallmaniae*	CBS 8575^T^	French Guiana	HQ283378	HQ283385
*Yamadazymatammaniensis*	CBS 8504^T^	USA	HQ283375	AF017243
*Yamadazymatenuis*	CBS 615^T^	Russia	HQ283371	U45774
*Yamadazymaterventina*	CBS 12510^T^	Italy	JQ247717	JQ247717
*Yamadazymathunbergiae*	JCM 36746^T^	Japan	LC805685	LC805685
*Yamadazymatrypodendroni*	CBS 8505^T^	Canada	FJ153212	AF017240
*Yamadazymatumulicola*	CBS 10917^T^	Japan	AB365463	AB365463
*Yamadazymaubonensis*	CBS 12859^T^	Thailand	NR_155998	AB759913
*Yamadazymavaughaniae*	CBS 8583^T^	French Guiana	HQ283364	HQ283381
*Yamadazymavrieseae*	CBS 10829^T^	Brazil	FJ755905	EU200785
*Yamadazyma* sp.	CLIB 1610	France	LN870324	LN870343
	14Y124	Japan	LC060705	LC060704
	GE19S08	Taiwan	FJ873424	FJ527153
	DMKU-RG72	Thailand	LC816131	LC816125
*Meyerozymaguilliermondii*	CBS 2030^T^	USA	KY104252	KY108542
*Babjeviellainositovora*	CBS 8006^T^	—	NR_111018	U45848

CBS, Westerdijk Fungal Biodiversity Institute, Utrecht, The Netherlands; DMKU, Culture Collection of the Department of Microbiology, Faculty of Science, Kasetsart University, Bangkok, Thailand; DSM, German Collection of Microorganisms and Cell Cultures GmbH, Braunschweig, Germany; TBRC, Thailand Bioresource Research Center, National Center for Genetic Engineering and Biotechnology, Pathumthani, Thailand; Strains marked with “T” are ex-type.

### ﻿Phylogenetic analyses

To infer phylogenetic relationships, newly generated ITS and LSU D1/D2 sequences from five isolates were combined with reference sequences retrieved from the GenBank database (Table 1). Taxon sampling was based on the frameworks established by Avesani et al. (2024) and Seike et al. (2024). Sequence alignment for each region was conducted using MAFFT v. 7.110 with the L-INS-i algorithm, which is optimized for accuracy (Katoh and Standley 2013). Alignments were inspected and edited using BioEdit v7.1.3.0 (Hall 1999), with manual refinements applied to improve character homology across taxa. The final ITS and LSU D1/D2 alignments were concatenated into a single dataset using PhyloSuite v. 1.2.3 (Zhang et al. 2020).

Phylogenetic analyses were performed using maximum likelihood (ML) and Bayesian inference (BI) methods. The most appropriate model of DNA substitution was selected using Modeltest v. 3.04 (Posada and Crandall 1998) with the Akaike information criterion (AIC). The model GTR + I + G was selected for both ML and BI analyses. ML analysis was conducted using RAxML v. 8.2.3 (Stamatakis 2014) with 1,000 bootstrap replicates. BI analysis was conducted using MrBayes v. 3.2.7a (Ronquist et al. 2012) with 50 million generations using the parameter settings described previously (Lu et al. 2024). Each tree was visualized with bootstrap support (BS) values ≥50% and Bayesian posterior probabilities (BPPs) ≥0.95 in FigTree v. 1.4.3 (Andrew 2016).

### ﻿Abbreviations

**GDMCC** Guangdong Microbial Culture Collection Center, Guangzhou, PR China;

**PYCC**Portuguese Yeast Culture Collection, Caparica, Portugal.

## ﻿Results

### ﻿Molecular phylogeny

**Figure 1.**
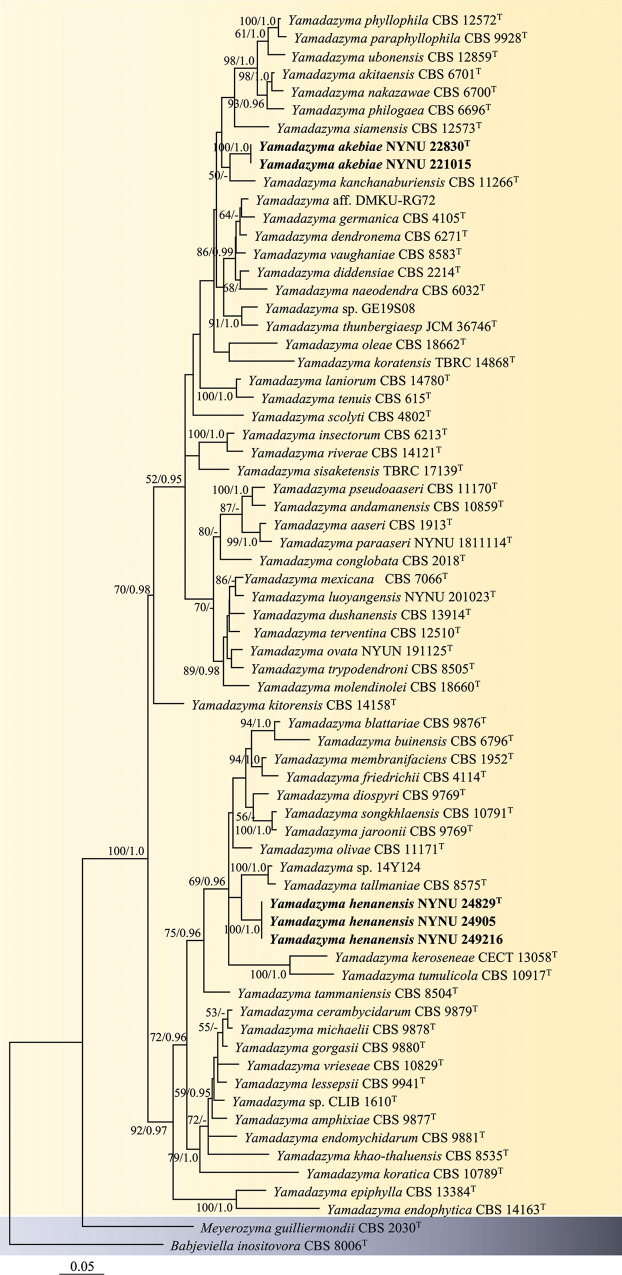
Phylogenetic tree of *Yamadazyma* inferred from maximum likelihood (ML) analysis based on a combined ITS and LSU D1/D2 dataset, with *Babjeviellainositovora*CBS 8006^T^ as the outgroup. Bootstrap support (BS) ≥ 50% and Bayesian posterior probabilities (BPPs) ≥ 0.95 are shown. Strains marked with “T” are ex-type. The strains from this study are highlighted in bold.

Phylogenetic reconstruction was based on the alignment of the concatenated ITS + LSU D1/D2 dataset, which included 69 ITS and 69 LSU D1/D2 sequences from 69 yeast strains representing 66 species. Phylogenetic trees inferred using both ML and BI methods exhibited similar topologies; therefore, only the tree derived from the ML analysis is presented (Fig. 1). The isolates from Guizhou and Hainan Provinces formed two distinct lineages in the phylogenetic tree (Fig. 1).

Strains NYNU 22830 and NYNU 221015, isolated from Guizhou Province, shared identical D1/D2 and ITS sequences, indicating they are conspecific. These strains are closely related to *Y.kanchanaburiensis*CBS 11266^T^ (Fig. 1), differing by four nucleotide (nt) substitutions (~0.8%) in the D1/D2 domain and 30 nt mismatches (~4.9%) in the ITS region. In comparison with other *Yamadazyma* species, they exhibit more than 9 nt substitutions (~1.7%) in the D1/D2 domain and 25 nt mismatches (~4%) in the ITS region. Based on these genetic differences, strains NYNU 22830 and NYNU 2487 are proposed as a new species, named *Yamadazymaakebiae* sp. nov.

Strains NYNU 24829, NYNU 24905, and NYNU 249216, isolated from Hainan Province, had identical D1/D2 and ITS sequences. These strains formed a well-supported clade within the genus *Yamadazyma* (Fig. 1) and were closely related to *Yamadazyma* sp. 14Y124 and *Y.tallmaniae*CBS 8575^T^, differing by 17–18 nt substitutions (~3.1–3.4%) in the D1/D2 domain and 29–33 nt mismatches (~4.7–5.2%) in the ITS region. Based on these genetic differences, these three strains are proposed as a new species, named *Yamadazymahenanensis* sp. nov.

### ﻿Taxonomy

#### ﻿ Yamadazyma akebiae

Taxon classificationFungiSaccharomycetalesDebaryomycetaceae

C.Y. Chai & F.L. Hui sp. nov.

**Figure 2.**
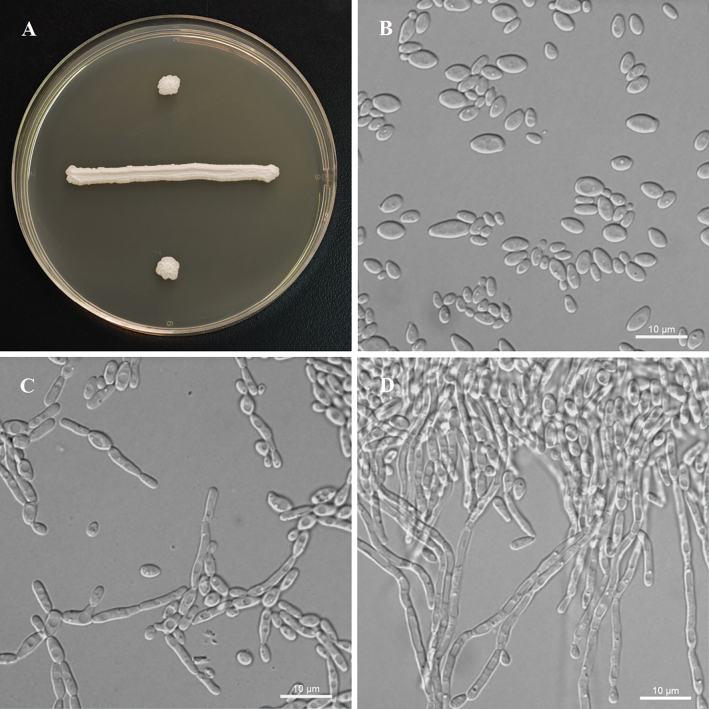
Morphology of *Yamadazymaakebiae* (ex-type, NYNU 22830). **A.** Colony on YM agar after 3 days at 25 °C; **B.** Budding cells on YM agar at 25 °C after 3 days; **C.** Simple pseudohyphae on CM agar after 3 days at 25 °C; **D.** Pseudohyphae with blastoconidia on CM agar after 7 days at 25 °C. Scale bars: 10 μm.

B6EF1452-F7F5-5906-8A3B-9CA7E910ABB2

858833

Fig. 2

##### Type.

China • Guizhou Prov.: Guiyang City, Guiyang Medicinal Botanical Garden, in the phylloplane of *Akebiatrifoliata*, August 2022, L. Zhang & F.L. Hui, NYNU 22830 (**holotype**GDMCC 2.303 preserved as a metabolically inactive state, ex-type PYCC 9930).

##### Etymology.

The specific epithet *akebiae* refers to *Akebia*, the plant genus from which the type strain was isolated.

##### Description.

After 7 days of growth on YM agar at 25 °C, the colonies appear white to cream-colored, lucid, and convex with irregular surfaces and margins. After 3 days of growth in YM agar at 25 °C, cells are ovoid to ellipsoid (2.5–3.9 × 4.1–7.4 μm) and occur singly or in pairs. Budding is multilateral. Pseudohyphae are formed, but true hyphae are absent in slide culture on cornmeal agar after 7 days at 25 °C. No asci or signs of conjugation are observed on YM agar, 5% ME agar, Fowell’s acetate agar, CM agar, or Gorodkowa agar after 6 weeks at 15 or 25 °C. Glucose and galactose are fermented, while sucrose, maltose, lactose, raffinose, trehalose, and d-xylose are not. Glucose, inulin, sucrose, galactose, trehalose, maltose, melezitose, methyl α-d-glucoside, cellobiose, salicin, l-sorbose, l-rhamnose, d-xylose, l-arabinose, d-arabinose, d-ribose, ethanol, glycerol, erythritol, ribitol, d-mannitol, d-glucitol, dl-lactate, succinate, citrate, d-gluconate, d-glucosamine, N-aetyl-d-glucosamine, and d-glucono-1, 5-lactone (delayed) are assimilated. No growth is observed with raffinose, melibiose, lactose, 5-keto-d-gluconate, methanol, galactitol, *myo*-inositol, 2-keto-d-gluconate, or d-glucuronate. In nitrogen-assimilation tests, growth is present in ethylamine, l-lysine, and cadaverine, while growth is absent in nitrate, nitrite, and creatine. Growth occurs at 35 °C, but not at 37 °C. No growth is observed in the presence of 10% NaCl with 5% glucose, 0.01% cycloheximide, or 1% acetic acid. Starch-like compounds are not produced. Urease activity and diazonium blue B reactions are negative.

##### Additional isolates examined.

China • Guizhou Province, Guiyang City, Guiyang Medicinal Botanical Garden, in the phylloplane of *Sapiumsebiferum*, August 2022, L. Zhang & F.L. Hui, strain number: NYNU 221015.

##### GenBank accession numbers.

Holotype GDMCC 2.303^T^ (ITS: OP566868; LSU D1/D2: OP566866); additional isolate NYNU 221015 (ITS: MZ318445; LSU D1/D2: MZ318422).

##### Notes.

Physiologically, *Y.akebiae* sp. nov. differs from its closely related species *Y.kanchanaburiensis* in its ability to assimilate inulin and dl-lactate and grow in vitamin-free medium (Table 2).

**Table 2.** Physiological characteristics of the new *Yamadazyma* species and their closely related taxa.

Characteristics	*Y.akebiae*	Y.kanchanaburiensis*	*Y.hainanensis*	Y.tallmaniae*
Fermentation of				
d-Glucose	+	w	–	+
Galactose	+	w	–	n
Assimilation of				
Inulin	+	–	w	n
Sucrose	+	+	–	+
Maltose	+	+	–	+
Melezitose	+	+	–	+
Methyl-α-D-glucoside	+	+	–	+
DL-Lactate	+	–	–	–
D-Glucosamine	+	s	–	+
Growth tests				
10%Nacl/5%glucose	–	n	–	+
Vitamin-free medium	+	–	+	n

+, positive reaction; –, negative reaction; d, delayed positive reaction; s, slow positive reaction; v, variable reaction; w, weak positive reaction; n, data not available. All data from this study, except * which were obtained from the original descriptions (Nakase et al. 2008; Groenewald et al. 2011).

#### ﻿ Yamadazyma hainanensis

Taxon classificationFungiSaccharomycetalesDebaryomycetaceae

C.Y. Chai & F.L. Hui sp. nov.

1926666C-49D0-57AB-BBF1-F7CD9FD80D50

858834

Fig. 3

##### Type.

China • Hainan Prov.: Wuzhishan City, Wuzhi Mountain, in the phylloplane of *Daemonoropsmargaritae*, August 2024, S.L. Lv, NYNU 24829 (**holotype**GDMCC 2.524 preserved as a metabolically inactive state, ex-type PYCC 10135).

##### Etymology.

The specific epithet *haianensis* refers to the geographic origin of the type strain, Wuzhi Mountain, Wuzhishan City, Hainan Province.

##### Description.

After 7 days of growth on YM agar at 25 °C, the colonies appear white to cream-colored, buttery, and smooth, with entire margins. After 3 days of growth in YM agar at 25 °C, cells are ovoid to ellipsoid (2.1–4.6 × 3.4–8.1 μm) and occur singly or in pairs. Budding is multilateral. Pseudohyphae are formed, but true hyphae are absent in slide culture on cornmeal agar after 7 days at 25 °C. No asci or signs of conjugation are observed on YM agar, 5% ME agar, Fowell’s acetate agar, CM agar, or Gorodkowa agar after 6 weeks at 15 or 25 °C. Fermentation is negative. Glucose, inulin (weak), galactose (weak), trehalose, cellobiose (weak), salicin (weak), d-xylose, l-arabinose, d-arabinose, d-ribose (delayed), ethanol, glycerol, erythritol, ribitol, d-mannitol, d-glucitol, succinate, citrate, d-gluconate, N-aetyl-d-glucosamine, and d-glucono-1, 5-lactone are assimilated. No growth is observed with sucrose, raffinose, melibiose, lactose, maltose, melezitose, methyl α-d-glucoside, l-sorbose, l-rhamnose, 5-keto-d-gluconate, methanol, galactitol, *myo*-inositol, dl-lactate, d-glucosamine, 2-keto-d-gluconate, or d-glucuronate. In nitrogen-assimilation tests, growth is present in ethylamine, l-lysine, and cadaverine, while growth is absent in nitrate, nitrite, and creatine. Growth is observed at 30 °C but absent at 35 °C. No growth is noted in the presence of 10% NaCl with 5% glucose, 0.01% cycloheximide, or 1% acetic acid. Starch-like compounds are not produced. Urease activity and diazonium blue B reactions are negative.

**Figure 3.**
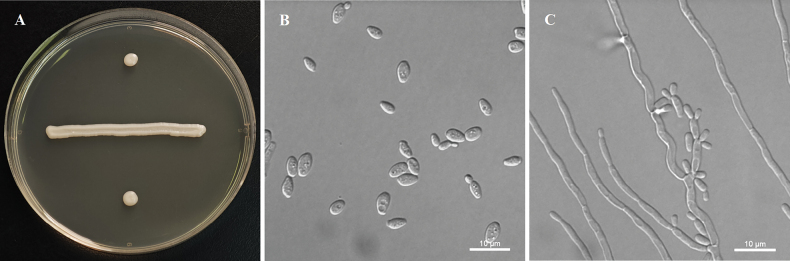
Morphology of *Yamadazymahainanensis* (ex-type, NYNU 24829). **A.** Colony on YM agar after 7 days at 25 °C; **B.** Budding cells on YM agar after 3 days at 25 °C; **C.** Pseudohyphae with blastoconidia on CM agar after 7 days at 25 °C. Scale bars: 10 μm.

##### Additional isolates examined.

China • Hainan Province, Wuzhishan City, Wuzhi Mountain, in the phylloplane of *Pollia* sp., August 2024, S.L. Lv, strain numbers: NYNU 24905, NYNU 249216.

##### GenBank accession numbers.

Holotype GDMCC 2.524^T^ (ITS: PQ568984; LSU D1/D2: PQ568981); additional isolates NYNU 24905 (ITS: MZ318442; LSU D1/D2: MZ318423) and NYNU 249216 (ITS: MZ318424; D1/D2 LSU: MZ318425).

##### Notes.

Physiologically, *Y.hainanensis* sp. nov. differs from its closely related known species, *Y.tallmaniae*, in several aspects (Table 2). *Y.hainanensis* sp. nov. is unable to ferment glucose, whereas *Y.tallmaniae* can. Additionally, *Y.hainanensis* sp. nov. is incapable of assimilating sucrose, maltose, melezitose, methyl-α-d-glucoside, or d-glucosamine, unlike *Y.tallmaniae*. Furthermore, *Y.tallmaniae* can grow in the presence of 10% NaCl with 5% glucose, while *Y.hainanensis* sp. nov. cannot.

## ﻿Discussion

Initially, the identification of *Yamadazyma* was primarily based on phenotypic traits, which resulted in its polyphyletic nature. However, with the advent of molecular phylogenetic techniques, more comprehensive approaches have been employed to address taxonomic challenges within the genus. For example, *Y.farinosa* was reclassified as *Millerozymafarinosa* and *Y.guilliermondii* as *Meyerozymaguilliermondii* based on concatenated LSU D1/D2 and SSU gene sequences (Kurtzman and Suzuki 2010). Further phylogenetic analysis using combined ITS and LSU D1/D2 sequences recognized *Yamadazyma* as a well-supported monophyletic clade (Groenewald et al. 2011). In accordance with the Melbourne Code (McNeill et al. 2012), which permits the assignment of related anamorphic and teleomorphic species to the same genus, additional anamorphic *Candida* species were subsequently reclassified into the genus *Yamadazyma*, thus broadening its diversity to encompass both teleomorphic and anamorphic forms.

In this study, two novel species—*Y.akebiae* sp. nov. and *Y.hainanensis* sp. nov.—are described based on a combination of molecular phylogenetic analyses and phenotypic characteristics. Phylogenetically, *Y.akebiae* sp. nov. is closely related to *Y.kanchanaburiensis*, forming a moderately supported clade. In contrast, *Y.hainanensis* sp. nov. forms a distinct branch clearly separated from other *Yamadazyma* species; however, a more robust phylogenetic placement for this species awaits further validation. The sequence divergences of the LSU D1/D2 and ITS regions between the new species and their closest relatives are below the similarity thresholds typically used to define species boundaries in ascomycetous yeasts (Kurtzman and Robnett 1998; Vu et al. 2016). Phenotypically, the two species share traits such as colony morphology, color, and cell shape, but can be differentiated from their closest relatives by distinct physiological and biochemical characteristics (Table 2). The integration of genetic, phylogenetic, and morphological data strengthens the reliability of species delimitation.

Yeast classification has traditionally relied on their ability to ferment sugars, typically measured by CO_2_ production (Kurtzman et al. 2011). Most ascomycetous yeasts, with few exceptions, are capable of sugar fermentation, whereas basidiomycetous yeasts generally are not. Interestingly, the three Chinese strains identified as *Y.hainanensis* sp. nov. were negative for sugar fermentation (Table 2), a rare feature within *Yamadazyma*. Only two other species, *Y.paraaseri* and *Y.andamanensis*, have been reported to lack this ability (Am-In et al. 2011; Gao et al. 2021; Avesani et al. 2024).

To date, 68 species of the genus *Yamadazyma* have been formally described, including the two introduced in this study (Avesani et al. 2024; Seike et al. 2024). Of these, 12 species have been recorded in China (e.g., *Y.diospyri*, *Y.paraphyllophila*, *Y.insectorum*, *Y.akitaensis*, *Y.olivae*, *Y.dushanensis*, *Y.luoyangensis*, *Y.ovata*, *Y.paraaseri*, *Y.triangularis*, *Y.akebiae*, and *Y.hainanensis*) (Gao et al. 2021; Cai et al. 2022). As one of the most biodiverse countries globally, China is likely home to many more undescribed *Yamadazyma* species. Ongoing surveys and taxonomic efforts are essential for a more complete understanding of species richness within the genus.

Numerous *Yamadazyma* species have been studied extensively, and some strains are used in agriculture and biotechnology (González-Gutiérrez et al. 2024; Lopes et al. 2018; Cai et al. 2022). While these yeasts are known to inhabit diverse ecological niches, environmental changes—especially those driven by climate change—are affecting both terrestrial and aquatic habitats (Jain et al. 2020). Such shifts may influence the global distribution and diversity of *Yamadazyma* species. Future research should therefore not only continue to document novel species and their distribution but also assess how climate variability may impact this ecologically and industrially important genus.

## Supplementary Material

XML Treatment for Yamadazyma akebiae

XML Treatment for Yamadazyma hainanensis

## ﻿Additional information

### Use of AI

No use of AI was reported.

### Author ORCIDs

Peng Wang https://orcid.org/0009-0001-8289-9640

Chun-Yue Chai https://orcid.org/0000-0003-0284-5560

Qiu-Hong Niu https://orcid.org/0000-0003-1695-7117

Feng-Li Hui https://orcid.org/0000-0001-7928-3055

### Data availability

All of the data that support the findings of this study are available in the main text.
